# Surgical Treatment for Empyema Thoracis: Prognostic Role of Preoperative Transthoracic Echocardiography and Serum Calcium

**DOI:** 10.3390/jpm12061014

**Published:** 2022-06-20

**Authors:** Pei-Yi Chu, Yu-Cheng Wu, Ya-Ling Lin, Hung Chang, Shih-Chun Lee, Tsai-Wang Huang, Yuan-Ming Tsai

**Affiliations:** 1 Division of Thoracic Surgery, Department of Surgery, Tri-Service General Hospital, National Defense Medical Center, Taipei 1140, Taiwan; chu.peiyi.88@gmail.com (P.-Y.C.); linlin988350@yahoo.com.tw (Y.-L.L.); hung@mail.ndmctsgh.edu.tw (H.C.); leesc001@yahoo.com.tw (S.-C.L.); chi-wang@yahoo.com.tw (T.-W.H.); 2 Department of Radiology, Tri-Service General Hospital, National Defense Medical Center, Taipei 11490, Taiwan; oliverudolf@hotmail.com; 3 Global Clinical Scholars Research Training (GCSRT) Program, Harvard Medical School, Boston, MA 02115, USA

**Keywords:** preoperative predictor, empyema thoracis, echocardiography, pulmonary arterial systolic pressure, hypocalcemia, postoperative outcome, video-assisted thoracoscopic surgery

## Abstract

Background: Empyema is a major cause of mortality and hospitalization. Symptoms include difficulty breathing and chest pain. Calcium plays an essential role in the physiology of the cardiovascular system. However, there is little evidence on the role of echocardiography and the serum calcium levels of patients undergoing video-assisted thoracoscopic surgery (VATS) for empyema. This study aimed to investigate the risk factors for postoperative mortality in patients with empyema who required surgery. Methods: This single-institution retrospective study compared the outcomes of VATS for thoracic empyema (in terms of survival and mortality) in 122 patients enrolled between July 2015 and June 2019. Results: This study examined patients with thoracic empyema. The majority of the patients were males (100/122, 81.9%). The in-hospital/30-day mortality rate was 10.6% (13 patients). The calcium levels were 7.82 ± 1.17 mg/dL in the survival group and 6.88 ± 1.88 mg/dL in the mortality group (*p* = 0.032). In the mortality group, the utilization of echocardiography and serum calcium levels independently contributed to the risk prediction more than clinical variables. Patients in our cohort exhibited elevated pulmonary artery systolic pressure (PASP) and hypocalcemia, which were associated with increased postoperative mortality. Conclusion: Elevated PASP and calcium levels at the low end of the normal range demonstrated significant prognostic value in predicting mortality in patients with thoracic empyema who required surgical intervention. Recognizing this potential is critical in order to obtain better outcomes.

## 1. Introduction

Despite advances in treatment, community-acquired pneumonia (CAP) is a common reason for medical consultation being sought in the emergency department (ED) [1]. Complicated parapneumonic effusion (CPPE) and thoracic empyema are the major complications of CAP necessitating surgical intervention, which can prolong hospital stay. Associated symptoms include chest pain, fever, and shortness of breath, and chest pain is the most frequent reason why patients present to the ED [2]. Cardiovascular events related to CAP have been reported as follows: decompensated heart failure, atrial fibrillation/flutter, and acute coronary syndromes [3]. Myocardial dysfunction is frequently associated with severe inflammation and septic shock [4]. Although cardiac complications are common in patients with CAP, the prevalence of cardiovascular dysfunction and its relationship with outcomes in patients with thoracic empyema remains unknown [5]. Prognostic factors such as CURB65 (new-onset confusion, urea, respiratory rate, systolic/diastolic blood pressure, and age) and the pneumonia severity index can be used to predict the 30-day mortality in patients with CAP who are admitted to hospital [6]. With the improvement of endoscopic techniques, video-assisted thoracoscopic surgery (VATS) is an excellent alternative to conventional open surgery for treatment. Although VATS offers superior outcomes for all ages and for advanced-stage thoracic empyema patients [7], mortality rates remain as high as 9% [8], and chest pain may be misdiagnosed, leading to post-surgery heart attack or injury. However, to date, only a few studies have investigated the risk factors and biomarkers for predicting the surgical outcomes of CPPE or empyema [9,10].

Calcium is essential for smooth muscle and cardiac muscle contractility as well as for hemodynamic stability. It aids in the stabilization of fibrinogen and platelets and contributes to several processes, including electrical conduction, coagulation, and hormone secretion [11,12]. The detrimental effects of worsening hypocalcemia in patients who have suffered from major trauma or undergone large-volume transfusion is well-known [13,14]. The link between low calcium concentration and critical illness, particularly cardiovascular dysfunction, has been established [12]. Previous studies have demonstrated that hypocalcemia is a laboratory abnormality in respiratory infections complicated by parapneumonic effusion [15]. Hypocalcemia is a common phenomenon among critically ill patients, ranging from 15 to 88% in adults [16]. However, the role of plasma calcium levels in patients with thoracic empyema has not been reported to date.

We recently showed that chest pain is the strongest prognostic factor after VATS decortication [10]. The effects of thoracic empyema on cardiac structures are attributed to systemic inflammatory activity, resulting in variations in coronary arterial tone and myocardial metabolic balance, but the role of echocardiography in patients with empyema presenting with chest pain is still uncertain [2]. Perioperative cardiac complications are the third leading cause of postoperative mortality. Echocardiography is a noninvasive, cost-effective, and reproducible diagnostic tool for evaluating left and right ventricular functions. Although there is insufficient clinical evidence to recommend its routine use for preoperative evaluation, some studies report echocardiography as a common test performed before major noncardiac surgery [17]. To date, studies on the relationships between echocardiographic findings and biomarkers are limited. Hence, this study aimed to explore the risk factors associated with post-surgery mortality for empyema, which may enable surgeons to predict and avoid postoperative complications and even to reduce mortality.

## 2. Materials and Methods

This retrospective study was conducted from July 2015 to June 2019 at a tertiary hospital in Taiwan. We included patients with thoracic empyema who were aged ≥18 years who had visited the ED and then had undergone VATS decortication. Exclusion criteria included patients with septic shock, immunocompromised status, cardiopulmonary distress, and long-term ventilator support and those without the required data. Because ionized calcium measurements were not routinely performed, the absence of these data did not serve as a reason for exclusion. We reviewed demographic data, comorbidities, clinical symptoms, echocardiographic data, and laboratory data, including blood and pleural fluid test findings, to evaluate mortality.

Pleural fluid was obtained via tube thoracostomy or thoracentesis. The definition of CPPE/empyema was based on Light’s criteria and the American College of Chest Physicians [18,19]. Preoperative blood tests performed within 1–3 days of scheduling surgery, including serum total calcium (normal, 8.6 to 10.2 mg/dL), potassium (normal, 3.5 to 5.1 mmol/L), and albumin levels (normal, 3.5 to 5.7 g/dL) were examined. The corrected calcium level (mg/dL) was calculated as follows: measured total calcium (mg/dL) + 0.8 × [4.0 − serum albumin levels (g/dL)]. The findings of transthoracic echocardiography were assessed during hospitalization before surgery to identify patients at risk of cardiac complications, focusing on pulmonary artery systolic pressure (PASP), aortic root diameters, and the left ventricular ejection fraction (LVEF). To rule out right ventricular outflow tract obstruction and pulmonary stenosis, patients underwent two-dimensional and color Doppler imaging. PASP was estimated using the modified Bernoulli equation with tricuspid regurgitant jet velocity plus the estimated right atrial pressure based on the inferior vena cava diameter and collapsibility. The LVEF was calculated using Simpson’s method. The outcomes measured were length of hospital stay and hospital mortality. Further, the 30-day postoperative mortality was used to assess and describe the risk and surgical prognosis. The study was approved by the author’s institutional review board (IRB No. A202105125).

## 3. Statistical Analysis

SPSS software version 22 (IBM, Armonk, NY, USA) was used for statistical analysis. Categorical variables are presented as frequencies, whereas continuous variables are presented as means ± standard deviations. Logistic regressions were used to identify potential predictive factors and evaluate the relationships between the variables with statistically significant differences between the two groups. Statistical significance was indicated by a two-sided *p*-value < 0.05.

## 4. Results

Among the 122 patients included in this study, 100 patients were male and 22 patients were female. In total, 13 (10.7%) of the 122 patients died within 30 days of the surgery. The mean age of the patients who died was higher than that of patients who survived, albeit not significantly (68.2 ± 13.1 vs. 60.8 ± 15.6 years; *p* = 0.10). The most common symptoms among patients presenting to the ED were dyspnea (51.6%), fever (50.0%), cough (34.4%), and chest pain (33.6%). Most of the patients (60.7%) had comorbidities, including coronary artery disease (27.0%), diabetes (22.9%), other malignancies (4.9%), and COPD (3.3%). Patients receiving the surgical approach had stage II empyema (118 cases) and stage III empyema (4 cases). Other variables such as body mass index, smoking history, side and stage of empyema, and the average operation time did not show statistically significant differences.

The average lengths of stay in the hospital for the survival group and mortality group were 25.6 days and 41.6 days, respectively (*p* = 0.019). Since the progression of thoracic empyema varies, the timing of surgical intervention and the need for preoperative echocardiography depend on the surgeon’s experience and judgment. In our study, 60.7% of the patients underwent echocardiography, including 56.9% in the survival group and 92.3% in the mortality group. The variables of the symptom of cough, comorbidity, average length of hospital stay, and echocardiography significantly differed between the mortality and survival groups (Table 1).

The laboratory data revealed that the patients who died had significantly higher serum potassium levels and lower serum calcium and albumin levels. Low albumin levels can affect the total serum calcium levels. Even after adjusting for albumin levels, the initial calcium levels remained low to low-normal in mortality patients compared to survival patients (8.25 ± 1.23 vs. 8.76 ± 1.18 mg/dL). The white blood cell (WBC) count, hemoglobin levels, creatinine levels, and C-reactive protein levels did not significantly differ between the two groups. Furthermore, no statistically significant differences were noted between the two groups in terms of the initial WBC count, protein levels, glucose levels, and lactate dehydrogenase levels in the pleural fluid (Table 2).

The echocardiographic parameters of patients in the mortality and survival groups are presented in Table 2. There were no statistically significant differences in aortic root diameters and left ventricular systolic function assessed with ejection fraction. Interestingly, echocardiography revealed statistically significant differences in PASP between the two groups. The factors described in the previous section were evaluated using a logistic regression analysis. The serum calcium levels (*p* = 0.047), serum albumin levels (*p* = 0.040), and PASP (*p* = 0.031) were found to be the independent risk factors for mortality (Table 3).

## 5. Discussion

In this study, we investigated the role of echocardiography and laboratory parameters in patients with thoracic empyema who underwent VATS. Our study found for the first time that postoperative mortality is related to echocardiography with elevated PASP and decreased serum calcium and albumin levels. Although there is growing speculation regarding the interactions between the pulmonary and cardiovascular systems, there is insufficient clinical evidence to recommend routine preoperative echocardiography. However, some studies have investigated the utility of echocardiography in various lung diseases [20] and reported it as a common test that is performed before major noncardiac surgery [17]. Although echocardiography is a quick, noninvasive way to evaluate ventricular function, the retrosternal position of the right ventricle limits its validity on the primary side of the respiratory infection [21]. Biteker et al. demonstrated that poor outcomes in patients with CAP might be associated with impaired right ventricular function with elevated N-terminal pro-B-type natriuretic peptide levels (NT-proBNP) [22]. Elevated PASP increases the static and pulsatile afterload of the right ventricle, leading to right ventricular enlargement and dysfunction, which is suggestive of pulmonary hypertension [23]. Recently, elevated PASP and increased mortality were revealed when PA pressure was measured by right heart catheterization or estimated by echocardiography [24]. Notably, PASP was significantly higher among patients in the mortality group who had thoracic empyema and who had undergone VATS, indicating that thoracic empyema may influence right ventricular function. Similar abnormal PASP values have also been reported to predict morbidity and mortality in patients with other acute conditions, but morbidity and mortality are frequently underestimated [25,26].

Although the mechanism underlying the link between increased PASP and thoracic empyema is unknown, our findings suggest that increased PASP could be a potential marker of disease activity and a predictor of prognosis in adult patients with thoracic empyema who undergo surgical treatment. Numerous studies have shown that inflammatory cytokines and stress are linked to hemodynamic and structural abnormalities in decompensated right ventricular failure, which is related to the activity of angiotensin II, natriuretic peptides, and reactive oxygen species [27,28]. Angiotensin II can influence calcium homeostasis and the production of cytokines such as tumor necrosis factor-α, interleukin-1, and interleukin-6 [29,30]. Recent studies have shown that hypocalcemia is associated with increased mortality in different populations, including medical, surgical, and trauma patients in adult and pediatric intensive care units [13,31]. We found that patients in the mortality group who had thoracic empyema and underwent VATS had increased PASP. Furthermore, lower serum calcium and albumin levels were significantly related to mortality.

Biomarkers can help with the diagnosis and prognosis of thoracic empyema. In patients with CAP, NT-proBNP, troponin I levels, creatinine levels, the mean platelet volume, the red blood cell distribution width, and albumin levels are useful markers for predicting mortality and disease severity [22]. The total serum and ionized calcium levels are the two most commonly tested parameters when assessing calcium levels. Although the analysis of ionized calcium (biologically active form) levels is more useful in clinical settings, its use is limited due to the time-consuming and costly procedure [32]. For every 1 g/dL decrease in the serum albumin level, the serum calcium level is reduced by 0.8 mg/dL [33]. In this study, the laboratory investigations revealed that the patients in the mortality group were hypocalcemic, with a mean calcium level of 6.88 ± 1.88 mg/dL (*p* = 0.032). Even after adjusting for albumin levels, the serum calcium levels in the mortality group remained lower than those in the survival group (8.25 ± 1.23 vs. 8.76 ± 1.18, respectively). Low albumin, a marker of malnutrition and severe sepsis, may be associated with mortality, and the relevance of hypocalcemia in patients with inflammation is still being debated. However, reduced intracellular calcium levels have been reported to be early predictors of mortality in critically ill surgical patients [12]. Calcium-phosphate metabolism has been linked to an increased risk of cardiovascular disease and mortality [34]. Some studies have also reported that hypocalcemia can cause electrocardiographic changes that are associated with an increased risk of mortality in patients with end-stage renal disease [35,36].

Several factors, including citrate in blood transfusion, increased phosphate concentration, renal failure, lower albumin levels, and vitamin D deficiency, may contribute to decreased calcium levels in patients with critical illnesses [12]. An imbalance in calcium levels may be involved in the release of cytokines in response to inflammatory stress. Notably, a higher number of patients in the mortality group underwent echocardiography and had elevated PASP, which could be the consequence of calcium-related vasoconstrictor responses to endogenous pulmonary vasoconstrictor substances. Studies on this topic are scarce; however, our study is particularly important, as we demonstrated significant differences between the survival and mortality groups. In patients with thoracic empyema undergoing VATS, using the uncorrected serum calcium level might help to assess the risk of poor prognosis and mortality. The prognostic implications of the inflammation-induced deregulation of calcium homeostasis for thoracic empyema and echocardiographic findings remain unclear. Although echocardiography has limitations in accurately measuring PASP in patients with advanced lung disease [37], appropriate knowledge regarding indications for echocardiography and a correct understanding of commonly encountered echocardiographic findings might help to manage patients in the perioperative period and avoid the risk of underestimating cardiovascular diseases such as cardiogenic shock or decompensated heart failure. Therefore, considering both PASP and calcium levels increased the prognostic value of the two markers. Patients with thoracic empyema with increased PASP and decreased calcium and albumin levels were at extremely high risk of poor postoperative outcomes. With an increase in life expectancy and the number of antibiotic-resistant cases, personalized medicine and individualized approaches can be evaluated based on institutional practice and risk factors to patients [38].

## 6. Limitations

Owing to its retrospective nature, this study has inherent limitations. The first was the lack of available data on a standardized and uniform management algorithm for the timing of echocardiography, blood examination, and calcium supplementation. Therefore, there may be a selection bias induced by only including individuals with preoperative data available. Calcium homeostasis can be disrupted by phosphate binders, vitamin D supplements, and other medications. The collection of more comprehensive data on pre- and postoperative ionized calcium levels would be advantageous. Furthermore, the analysis of phosphate and ionized calcium levels might help to establish more definitive conclusions. Second, many patients were already pretreated and secondarily referred by primary physicians or other hospitals, and many initial details are now impossible to retrieve. Third, our findings may be compromised due to the inclusion of a patient population that was predominantly from a single center. Additionally, we cannot exclude the possibility of residual confounders despite adjusting the regression models for a variety of potential confounders. For example, the lack of information about in-hospital cardiac events might contribute to PASP. To the best of our knowledge, this is the first study to investigate the relationship among echocardiographic findings, serum calcium levels, and outcomes while controlling for common variables. It would be interesting to see how mortality, echocardiography findings, and calcium homeostasis are affected.

## 7. Conclusions

In patients with thoracic empyema, PASP may be elevated and serum calcium and albumin levels in low ranges are associated with an increased risk of mortality. More patients must be studied in order to determine the prognostic significance of PASP and calcium supplements in patients with thoracic empyema.

## Figures and Tables

**Table 1:** Patient characteristics and comparison between survival and mortality patients.

Demographic Data	Survivors (*n* = 109)	Mortality (*n* = 13)	*p* Value
Age (mean ± SD)	60.8 ± 15.6	68.2 ± 13.1	0.100
Male: Female	88:21	12:1	0.194
BMI	22.9 ± 4.5	22.1 ± 2.8	0.537
Smoking	51 (46.8%)	7 (53.8%)	0.633
Pack-years	33.1 ± 13.1	30.0 ± 6.3	0.356
Symptoms			
Cough	42 (38.5%)	0 (0.0%)	<0.001
Fever	56 (51.4%)	5 (38.5%)	0.398
Pain	38 (34.9%)	3 (23.1%)	0.378
Dyspnea	57 (52.3%)	6 (46.2%)	0.678
Comorbidity	63 (57.8%)	11 (84.6%)	0.031
Coronary arterial disease	30 (27.5%)	3 (23.1%)	0.736
Type 2 DM	23 (21.1%)	5 (38.5%)	0.162
COPD	4 (3.7%)	0 (0.0%)	0.482
Other malignancy	14 (12.8%)	2 (15.4%)	0.798
Stage 2/3	105/4 (96.3%/3.7%)	13/0 (100%/0.0%)	0.487
Side (right)	68 (62.4%)	6 (46.2%)	0.261
Echocardiography	62 (56.9%)	12(92.3%)	0.001
Average operation time (min)	98.29 ± 44.85	87.62 ± 42.76	0.417
Average length of hospital stay (days)	25.66 ± 23.01	41.62 ± 21.18	0.019

BMI: body mass index; DM: diabetes mellitus; COPD: chronic obstructive pulmonary disease.

**Table 2:** Distribution of laboratory observations and echocardiographic data between survival and mortality patients.

Variables			Survivors (*n* = 109)	Mortality (*n* = 13)	*p* Value
Blood	WBC(/µL)		16,126 ± 8206	12,894 ± 5530	0.309
		Neut(%)	81.7 ± 9.7	83.4 ± 7.9	0.407
		Lym(%)	10.1 ± 6.8	9.4 ± 5.7	0.975
	Hemoglobin		11.45 ± 2.18	11.54 ± 2.98	0.102
	Creatinine		1.37 ± 0.13	1.45 ± 0.43	0.493
	Calcium		7.82 ± 1.17	6.88 ± 1.88	0.032
	Potassium		3.89 ± 0.61	4.22 ± 0.88	0.042
	Albumin		2.78 ± 0.54	2.51 ± 0.30	0.035
	CRP		18.22 ± 9.96	16.35 ± 9.98	0.781
	Corrected calcium		8.76 ± 1.18	8.25 ± 1.23	0.201
Pleural effusion	WBC		7213 (0–66,900)	11,091 (80–78,422)	0.305
	Total protein		1.5 (0.4–7.2)	1.5 (1.2–5.2)	0.073
	LDH		1139 (0–12,941)	3271 (0–5045)	0.972
	Glucose		104 (0–682)	130 (4–305)	0.530
Echocardiography	PASP (mmHg)		32.8 ± 11.9	41.8 ± 15.1	0.038
	Aortic root diameters (mm)		33.0 ± 5.7	34.4 ± 3.6	0.212
	LVEF (%)		62.3 ± 11.5	59.9 ± 15.8	0.444

WBC: white blood cell; CRP: C-reactive protein; LDH: lactate dehydrogenase; PASP: pulmonary artery systolic pressure; LVEF: left ventricular ejection fraction.

**Table 3:** Multivariate logistic regression models for independent predictors of mortality.

Variables	Odds Ratio	Std. Error	95% Confidence Interval	*p* Value
Comorbidity	2.347	1.077	0.284–19.375	0.428
Average length of hospital stay (days)	0.991	0.019	0.956–1.028	0.629
Calcium	2.082	0.369	1.010–4.289	0.047
Potassium	1.512	0.616	0.452–5.062	0.502
Albumin	3.625	0.630	1.054–12.461	0.040
Echocardiography	9.097	1.059	1.142–72.446	0.037
PASP	0.878	0.060	0.780–0.988	0.031

PASP: pulmonary artery systolic pressure.

## Data Availability

The data presented in this study are available on request from the corresponding author. The data are not publicly available due to ethical restrictions.
